# Autoimmune Lymphoproliferative Syndrome: A Rare Cause of Disappearing HDL Syndrome

**DOI:** 10.1155/2016/7945953

**Published:** 2016-08-08

**Authors:** Swetha Sriram, Avni Y. Joshi, Vilmarie Rodriguez, Seema Kumar

**Affiliations:** ^1^Division of Pediatric Endocrinology, Mayo Clinic, Rochester, MN 55902, USA; ^2^Division of Pediatric Allergy and Immunology, Mayo Clinic, Rochester, MN 55902, USA; ^3^Division of Pediatric Hematology-Oncology, Mayo Clinic, Rochester, MN 55902, USA

## Abstract

The term disappearing HDL syndrome refers to development of severe high density lipoprotein cholesterol (HDL-C) deficiency in noncritically ill patients with previously normal HDL-C and triglyceride levels. Autoimmune lymphoproliferative syndrome (ALPS) is a disorder of the immune system due to an inability to regulate lymphocyte homeostasis resulting in lymphadenopathy and hepatosplenomegaly. We describe a 17-year-old boy who was evaluated in the lipid clinic for history of undetectable or low HDL-C and low density lipoprotein cholesterol (LDL-C) levels. Past medical history was significant for ALPS IA diagnosed at 10 years of age when he presented with bilateral cervical adenopathy. He was known to have a missense mutation in one allele of the FAS protein extracellular domain consistent with ALPS type 1A. HDL-C and LDL-C levels had been undetectable on multiple occasions, though lipids had not been measured prior to the diagnosis of ALPS. He had been receiving sirolimus for immunosuppression. The HDL-C and LDL-C levels correlated with disease activity and improved to normal levels during times when the activity of ALPS was controlled. This case highlights the importance of considering ALPS as a cause of low HDL-C and LDL-C levels in a child with evidence of lymphoproliferation.

## 1. Introduction 

Low levels of high density lipoprotein (HDL) cholesterol (<40 mg/dL) is the most common lipid abnormality seen in children (13%–15%) [1, 2]. The most common cause of low HDL cholesterol is obesity and insulin resistance and HDL cholesterol tends to be usually above 20 mg/dL in most children. Testing for genetic disorders is often considered in children with extremely low levels of HDL cholesterol (<20 mg/dL) and is associated with significant costs. Knowledge of secondary causes of extremely low HDL cholesterol levels is important in order to avoid costly genotyping and inappropriate genetic counseling and due to a different prognosis. Here we describe a child with autoimmune lymphoproliferative syndrome (ALPS) IA who had undetectable HDL cholesterol and low density lipoprotein (LDL) cholesterol levels on multiple occasions and in whom lipid levels correlated with disease activity and improved with treatment for ALPS.

## 2. Case Report 

A 17-year-old boy was referred to the pediatric lipid clinic for evaluation of low HDL cholesterol and low LDL cholesterol. His past medical history was significant for autoimmune lymphoproliferative syndrome (ALPS) type 1A that had been diagnosed in October 2007 at age of 10 years following one year history of bilateral cervical lymphadenopathy. CT scan of the abdomen had revealed generalized adenopathy with numerous prominent lymph nodes in the axilla bilaterally, supraclavicular, retroperitoneal, and inguinal areas and throughout the mesentery. In addition, splenomegaly (18.3 cm pole to pole) was also noted. Biopsy of the cervical lymph node revealed reactive hyperplasia and flow cytometry of peripheral blood revealed 23% double negative T cells and 11% alpha beta double negative T cells. Genetic testing for ALPS was performed and he was found to have a missense mutation, 621 (T>C) (C127R), in one allele of the FAS protein extracellular domain consistent with ALPS type 1A.

A year after his diagnosis, he was started on mycophenolate mofetil and also received a two-month course of prednisone for progressive lymphadenopathy. Mycophenolate was discontinued and sirolimus was started three years after diagnosis due to continued lymphadenopathy. Lipid profile was checked for the first time nearly three years after diagnosis of ALPS and three months after starting sirolimus due to known effect of sirolimus on increasing triglycerides. HDL cholesterol was noted to be low at 31 mg/dL, with repeat levels of 46 and 60 mg/dL (low < 40 mg/dL, borderline low 40–60 mg/dL, Table 1). LDL cholesterol and triglycerides were normal. Two years after the initial lipid profile, LDL cholesterol and HDL cholesterol became undetectable on multiple occasions over a period of six months. Triglyceride levels were only slightly elevated on two occasions (116 mg/dL and 148 mg/dL; normal < 90 mg/dL; borderline high 90–129 mg/dL and high ≥ 130 mg/dL) but were normal when HDL and LDL cholesterol levels were undetectable. Patient was not obese or diabetic at any of the times when the HDL cholesterol was undetectable with body mass index being between the 50th and 75th age and gender specific percentile. Patient was never on any medications such as peroxisome proliferator-activated receptor agonists, isotretinoin, protease inhibitors, nonselective beta blockers, and androgenic steroids which are known to reduce HDL cholesterol. Patient did not have any malabsorption, liver disease, or other chronic or acute inflammatory disorder.

**Figure 1:**
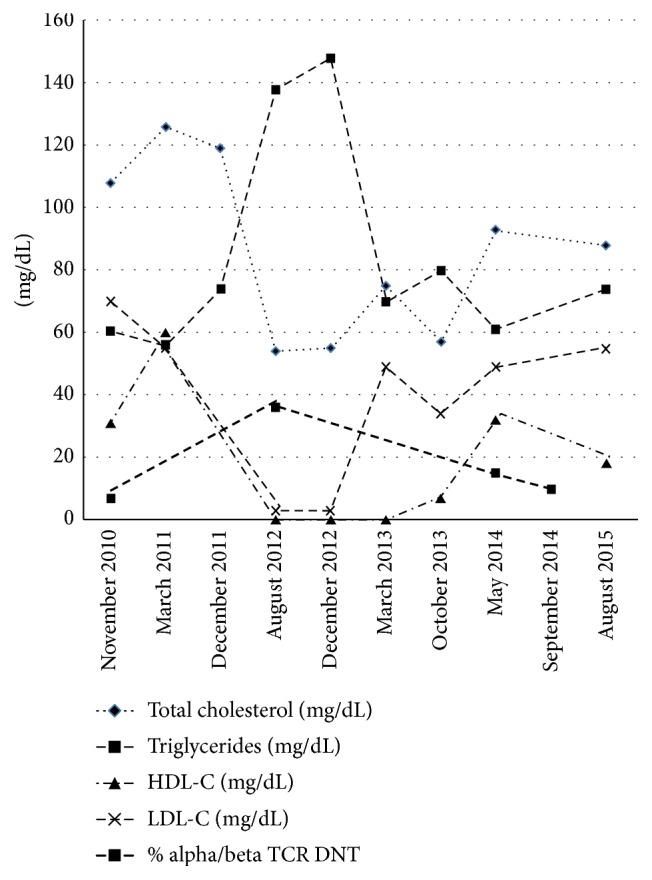
Relationship between lipid parameters and disease activity marker % alpha/beta TCR/DNT.

HDL cholesterol and LDL cholesterol levels improved with decrease in disease activity as measured by % alpha/beta TCR/DNT levels (Table 1 and Figure 1). While HDL cholesterol levels have improved but still continue to be low, LDL cholesterol levels have normalized. There was a significant negative correlation between disease activities as assessed by % alpha/beta TCR/DNT levels and total cholesterol (*r* = −0.9734, *p* = 0.038) and LDL cholesterol levels (*r* = −0.9962, *p* = 0.049). Patient continues to do well with decrease in lymphadenopathy on sirolimus. He continued to have splenomegaly despite the immunosuppression and underwent splenectomy at age 15 years of age in order to make contact sports safer without risk for splenic rupture.

## 3. Discussion

The term disappearing HDL syndrome coined by Goldberg and Rader refers to cases of severe HDL cholesterol deficiency in noncritically ill patients long before clinical and biochemical features of the underlying disease becomes evident [3]. Once the offending cause is removed, HDL cholesterol levels typically return to their previous values [3].

We report a child with ALPS 1A, a disorder of immune dysregulation who was noted to have undetectable HDL cholesterol and undetectable LDL cholesterol on multiple occasions. The lipid levels in this child correlated with disease activity and improved with therapy as has been reported by other groups [4, 5].

Primary causes for low HDL cholesterol include genetic disorders due to mutations in the ATP-binding cassette transporter A1, apolipoprotein A-I, and lecithin-cholesterol acyltransferase (LCAT) [6]. The most common secondary causes for significantly low HDL cholesterol levels (<20 mg/dL) include hypertriglyceridemia, HIV, renal disease, acute inflammation, and chronic inflammatory diseases, such as rheumatoid arthritis and systemic lupus erythematosus, and medications such as isotretinoin, sirolimus, protease inhibitors, nonselective beta blockers, and androgenic steroids [7, 8]. Similarly, primary causes for low LDL cholesterol levels include genetic disorders, namely, abetalipoproteinemia due to a mutation in the gene encoding microsomal transfer protein and hypolipoproteinemia due to mutations that may occur at multiple genetic loci including that encoding apo B [9]. Low LDL cholesterol can also be seen in clinical states, such as occult malignancy, malnutrition, critical illness, and chronic liver disease [10–12].

Our report of undetectable HDL cholesterol and undetectable LDL cholesterol in a patient with ALPS IA highlights the need for health care providers to be aware of this condition as a secondary cause of low HDL cholesterol and low LDL cholesterol. The low HDL cholesterol in this disorder does not require any further workup in terms of genetic testing and intervention with physical activity or medications. Autoimmune lymphoproliferative disorder/syndrome (ALPS) is a disorder of the immune dysregulation due to an inability to regulate lymphocyte homeostasis through the process of lymphocyte apoptosis. The consequences of this lymphoproliferative process are manifested by lymphadenopathy, hepatomegaly, splenomegaly, and an increased risk of lymphoma as well as autoimmune disease [13, 14]. Soluble IL-10, FasL, IL-18, and vitamin B12 are known biomarkers for presence of FAS mutation ALPS, but there are no biomarkers to help assess disease severity once treatment with sirolimus is initiated [15].

Low HDL cholesterol in children with ALPS has been described previously [4, 5]. Similar to our case, the HDL cholesterol levels in patients in a previous report also improved with therapy [4]. Decreased plasma LCAT activity is believed to be the likely pathophysiologic mechanism underlying low levels of HDL cholesterol in patients with ALPS as Moraitis and colleagues demonstrated that the HDL subpopulation distribution on native-native 2 gel analysis in a patient with large B cell lymphoma, another lymphoproliferative disorder and disappearing HDL syndrome, was noted to be similar to the profile found in familial LCAT deficiency with predominance of pre-*β* HDL and *α*4 HDL particles, absence of larger spherical particles (*α*1–3), and abnormally low plasma cholesteryl ester [5]. Additionally, LCAT activity was found to be markedly decreased in 3 patients with disappearing HDL cholesterol secondary to large B cell lymphoma and diffuse large cell lymphoma compared with healthy controls prior to treatment and increased following chemotherapy. Low LCAT activity was associated with low HDL cholesterol levels and the increase in LCAT activity following chemotherapy correlated with an increase in HDL cholesterol in all three patients studied [5].

High levels of interleukin-10 (IL-10) are the likely causative factor for low HDL cholesterol levels in children with ALPS [5]. An inverse correlation between IL-10 and HDL cholesterol levels was noted in a study of 93 patients with ALPS [5]. Patients with disappearing HDL cholesterol due to B cell lymphoma diffuse large cell lymphoma and ALPS have been noted to have high IL-10 levels and low HDL cholesterol prior to chemotherapy [5]. A decrease in IL-10 levels after chemotherapy was associated with an increase in HDL cholesterol levels [5]. A direct causal link between IL-10 and lipoprotein levels was also suggested from a randomized placebo controlled clinical trial of recombinant human IL-10 in patients with psoriatic arthritis. HDL cholesterol levels decreased rapidly by mean of 76% to near-undetectable levels and LDL cholesterol levels decreased by more than half within a week of initiating subcutaneous recombinant human IL-10 injections. Additionally, lipoprotein levels returned to baseline four weeks after discontinuation of IL-10 therapy [16].

The mechanisms by which highly elevated IL-10 lead to low HDL cholesterol and low LDL cholesterol remains to be explored. Expression of detectable amounts of IL10R1, the subunit of the IL-10 receptor that confers specificity for IL-10 has not been noted in hepatocytes [17]. It is possible that an alternative low-affinity IL-10 receptor in hepatocytes might play a role in the setting of extremely high IL-10 levels. IL-10 may also interact with canonical IL-10 receptors in nonhepatocyte liver cells such as tissue macrophages, endothelial cells, and hepatic stellate cells that might in turn signal to hepatocytes. Other mechanisms that might account for the HDL cholesterol lowering effect of IL-10 could be decreased apo A-I synthesis and secretion in response to IL-10 in the intestine and increased catabolism of HDL in response to highly elevated IL-10 [5].

## 4. Conclusion

ALPS should always be considered in the differential diagnosis when a child is noted to have low HDL cholesterol levels with evidence of lymphoproliferation. Testing for genetic causes of low HDL cholesterol should be considered only after exclusion of secondary causes. HDL cholesterol can be useful biomarker of both disease activity and compliance in children with ALPS.

## Figures and Tables

**Table 1:** Serial lipid measurements in the patient.

Parameter (mg/dL)	Nov 10	Dec 10	Mar 11	Dec 11	Aug 12	Dec 12	Mar 13	Oct 13	May 14	Aug 15
Total cholesterol	108	122	126	119	54	55	61	57	93	88
Triglycerides	60.5	66	56	74	138	148	116	80	61	74
HDL cholesterol	31	46	60	ND	**<5**	**<5**	**<5**	7	32	18
Calculated LDL cholesterol	70	63	55	ND	NQ	NQ	NQ	34	49	55

ND: not done and NQ: unable to quantify.
